# Cloning and expression of lin-28 homolog B gene in the onset of puberty in Duolang sheep

**DOI:** 10.5713/ajas.18.0276

**Published:** 2018-10-26

**Authors:** Feng Xing, Chaoyang Zhang, Zhengquan Kong

**Affiliations:** 1 College of Animal Science, Tarim University, Alar, XinJiang 843300, China; 2 Key laboratory of Tarim, Animal Husbandry Science and Technology, XinJiang Production & Construction Corps, Alar, Xinjiang 843300, China

**Keywords:** Sheep, Puberty, Expression Profile, *Let-7* miRNA

## Abstract

**Objective:**

Recent studies have demonstrated that lin-28 homolog B (*LIN28B*)/miRNA let-7 (*let-7*) plays a role in the regulation of pubertal onset in mammals. However, the role of *LIN28B*/*let-7* in the onset of ovine puberty remains unknown. We cloned the Duolang sheep *Lin28B* cDNA sequence, detected the expression change of *LIN28B*, *let-7a* and *let-7g* in hypothalamus, pituitary and ovary tissues at three different pubertal stages.

**Methods:**

The reverse transcriptase polymerase chain reaction (RT-PCR) was used to clone the cDNA sequence of *LIN28B* gene from Duolang sheep and the bioinformatics methods were applied to analyze the amino acid sequence of *LIN28B* protein. The mRNA expression levels of the *LIN28B* gene at different pubertal stages were examined by real time RT-PCR.

**Results:**

*LIN28B* cDNA of Duolang sheep was cloned, and two transcripts were obtained. The amino acid sequence of transcript 1 shares 99.60%, 98.78%, and 94.80% identity with those of goat, wild yak and pig, respectively. Strong *LIN28B* mRNA expression was detected in the hypothalamus, pituitary, ovary, oviduct and uterus, while moderate expression was found in the liver, kidney, spleen and heart, weak expression was observed in the heart. No expression was found in the lungs. Quantitative real-time PCR (QPCR) and western-blot analysis revealed that the *LIN28B* was highly expressed in the hypothalamus and ovary at prepuberty stages, and this expression significantly decreased from the prepuberty to puberty stages (p<0.05). Markedly increased levels of mRNA expression were detected in the pituitary from prepuberty to puberty (p<0.05) and then significantly decreased from puberty to postpuberty (p<0.05). The expression levels of *let-7a* and *let-7g* showed no significant changes among different pubertal stages (p>0.05).

**Conclusion:**

These results provided a foundation for determining the functions of *LIN28B*/*let-7* and their role in the onset of sheep puberty.

## INTRODUCTION

Puberty is a key stage in which animals attain fertility for the first time and involves a complex series of events that are governed by the activation of the hypothalamic-pituitary-gonadal axis. The age of puberty is a moderately heritable trait, with an average heritability of 0.32 [1,2]; normal or disturbed pubertal development is mainly determined by genetic factors [3,4]. The onset of puberty is closely related to changes in the transcription and expression levels of related genes, and lin-28 homolog B (*LIN28B*) is a particularly important gene associated with the onset of puberty [5–7].

The *LIN28* family includes two homologous members, *LIN28A* and *LIN28B*, each with a similar domain structure and function [8–10]. *LIN28B*, which contains a cold shock domain (CSD) and a retroviral-type CCHC zinc finger motif, was first cloned and identified as an over-expressed factor in hepatocellular carcinoma cells [10]. *LIN28B* blocks miRNA let-7 (*let-7*) expression, whereas *let-7* negatively regulates *LIN28B* expression by binding to the 3′UTR of *LIN28B*, thereby establishing a double negative feedback loop. However, the expression patterns of *LIN28B* compared to those of *let-7* are not completely reciprocal [11–14].

In rodents, expression of *LIN28B* and *let-7* in the hypothalamus-pituitary-gonad (HPG) tissues has been widely studied, the results show that *LIN28B* is expressed at a high level in the hypothalamus and testes, and that this expression is reversed in the ovary [15–17]. In humans, *LIN28B* is widely expressed in adult normal tissues, and its expression level is highest in the testis and placenta [10]. In adult rats, the *LIN28B* mRNA expression level is higher in the testis, placenta and hypothalamus, whereas no expression is detected in the ovary [14].

Numerous studies have reported the vital correlation between *LIN28B*/*let-7* and the onset of puberty in mice, monkeys, among other species, but there are few studies on the role of *LIN28B*/*let-7* in the onset of ovine puberty [13].

Duolang sheep are a typical representative of an early maturing breed in Xinjiang, and its age of puberty is only 3–4 months under the same ecological conditions [18]. In the present study, the *LIN28B* cDNA sequence from Duolang sheep was cloned and sequenced, and its expression profile was characterized in ten tissues. The expression levels of *LIN28B*, *let-7a* and *let-7g* were detected in the hypothalamus, pituitary and ovary at three different pubertal stages.

## MATERIALS AND METHODS

This work was conducted in accordance with the specifications of the Ethics Committee of Tarim University of Science and Technology.

### Animals and tissue collection

Fifteen female Duolang sheep were housed, including five prepubertal, five pubertal, and five postpubertal sheep, all of which were obtained from the same line and reared under similar conditions on a farm in Xinjiang, China. We determined pubertal sheep in a study of female sheep by detecting estrus and changes in the appearance of the vulva [19,20]. Sheep were deeply anesthetized by intravenous administration of 3% pentobarbital sodium (30 mg/kg; Solarbio, P8410, Beijing, China) and sacrificed by exsanguination at a healthy physiological stage. The hypothalamus, pituitary, ovary, oviduct, uterus, liver, heart, kidney, lung and spleen were surgically removed and snap frozen in liquid nitrogen for mRNA extraction.

### Total RNA isolation and cDNA synthesis

Total RNA was isolated from the hypothalamus, pituitary, ovary, oviduct, uterus, liver, heart, kidney, lung and spleen by using TRIzol reagent (Invitrogen, Carlsbad, CA, USA) according to a standard extraction protocol. The integrity of total RNA was evaluated on 1.5% agarose gels containing formaldehyde and ethidium bromide. First-strand cDNA was synthesized as follows: a mixture of 2 μg of total RNA, 1 μL of the oligo(dT)_15_ primer and 1 μL of dNTPs (2.5 mM each) was heated for 5 min at 65°C and chilled on ice; then, 4 μL of 5× first-strand buffer, 0.5 μL of RNase Inhibitor (40 μL, TaKaRa, Dalian, China) and 1 μL of M-MLV enzyme (200 U/μL, TaKaRa, China) were added, and the reaction was incubated for 50 min at 42°C, followed by 5 min at 95°C to inactivate the reverse transcriptase. The reaction was finally stored at −20°C for subsequent gene cloning and mRNA expression analysis.

### Cloning and sequencing of sheep *LIN28B* cDNA

*LIN28B* mRNA sequences of sheep and cattle (GenBank: JQ277700.1, XM_012182382.2 and XM_024997174.1, respectively) were used to design the primers for cloning (Table 1), and *LIN28B* cDNA was obtained from total RNA isolated from the ovary tissues of Duolang sheep. The polymerase chain reaction (PCR) was performed in a 25 μL total volume, containing 1 μL of first-strand cDNA template, 0.5 μL of each primer (10 μM), and 12.5 μL of PCR mix (TaKaRa, China). Reverse transcriptase-PCR (RT-PCR) with primers (Table 1) was performed at 95°C for 5 min, followed by 35 cycles of 95°C for 30 s, annealing for 30 s and 72°C for 1 min, followed by one cycle of 72°C for 5 min. The amplified PCR product was purified and ligated into pMD18-T (TaKaRa, China) and then transformed into competent *Escherichia coli* DH5α cells. At least four positive clones were sequenced with an ABI 3730 sequencer (Applied Biosystems, Foster City, CA, USA).

### Reverse-transcription polymerase chain reaction

The RT-PCR was performed to examine the *LIN28B* expression profile in ten tissues, including the hypothalamus, pituitary, ovary, oviduct, uterus, liver, heart, kidney, lung and spleen. PCR amplifications were performed in a 25 μL volume containing 1 μL of first-strand cDNA template, 0.5 μL of each primer (10 μM), and 12.5 μL of PCR mix (TaKaRa, China). For tissue expression analysis, beta-actin (*ACTB*) was used as the internal control and amplified with the specific primers *ACTB*-F and *ACTB*-R (Table 1). PCR products for *ACTB* and *LIN28B* were run separately on 1.5% agarose gels. The identity of each amplified fragment was confirmed by direct sequencing on an ABI 3730 sequencer (Applied Biosystems, USA).

### Expression of *LIN28B*, *let-7a* and *let-7g* in tissues by quantitative real-time polymerase chain reaction

The expression of *LIN28B*, *let-7a*, and *let-7g* in the hypothalamus, pituitary and ovary tissues of Duolang sheep at three different pubertal stages was assessed by quantitative real-time PCR (qPCR).

For *LIN28B* mRNA expression, *ACTB* was selected as the reference gene, and its quantification cycle was used to normalize the mRNA levels of the target gene. Primers were designed to span exon–exon boundaries to ensure specificity, which was further ensured according to dissociation curve, analysis and sequencing. The following primer sequences were used for *LIN28B* and *ACTB*: *LIN28B* F: 5′-aggaagcgaaagaagaccca -3′, R: 5′-gcacttctttggctgaggag -3′; and *ACTB* F: 5′-ttccagccttccttcctg -3′, R: 5′-ccgtgttggcgtagaggt -3′.

For *let-7a* and *let-7g* quantification, cDNA was synthesized by using a One-Step miRNA RT Kit. The mature sequences of *let-7a* and *let-7g* were obtained by using miRBase, and these sequences were used to design the primers. The forward primers for *let-7a* and *let-7g* were designed, while the reverse primers for *let-7a* and *let-7g* were included in the Sino Gene One-step miRNA RT Kit; U6 was selected as a reference gene. The following primer sequences were used for *let-7a*, *let-7g* and *U6* snRNA: *let-7a*-F: 5′-gctgaggtagtaggttgtatagtt -3′; *let-7g*- F: 5′-cgtgaggtagtagtttgtacatgt -3′; and U6 - F: 5′-ctcgcttcggca gcacatat -3, R: 5′-aacgcttcacgaatttgcgt -3′.

PCR was performed in a total volume of 20 μL, containing 1 μL of first-strand cDNA template, 7.5 μL of 2× SG Green qPCR Mix, and 0.25 μL of each primer. PCR was performed under the following conditions: 95°C for 10 min, followed by 40 cycles at 95°C for 20 s and 60°C for 30 s, then at 95°C for 15 s, 60°C for 30 s, and 95°C for 15 s. Each sample was run three times on three separate assays. The expression levels of *LIN28B*, *let-7a*, and *let-7g* were calculated by the 2^−ΔΔCT^ method [21].

### Western blot analysis

Total protein extraction: the tissue was frozen in liquid nitrogen, and 100 mg of the tissue was added to 500 μL of RIPA lysate and mixed well, and then, the mixture was placed on ice for 30 min and homogenized; the mixture was then centrifuged at 12,000 rpm and 4°C for 15 min, and the supernatant was collected.

Protein concentrations were quantified with the Bicinchoninic Acid Protein Assay Kit (CW Biotech, Beijing, China), and the samples were incubated overnight with one of the following primary antibodies at 4°C: anti-Lin28B antibody (1:1,000, ab46020, Abcam, Boston, MA, USA) or anti-β-actin antibody (1:1,000).

The secondary antibodies were horseradish peroxidase-conjugated goat anti-rabbit antibodies (1:5,000). An enhanced chemiluminescence kit (Millipore, Billerica, MA, USA) was used for detection.

### Statistical analysis

For qPCR analysis, the mRNA levels of *LIN28B*, *let-7a*, and *let-7b* were expressed as the means±standard error, and the means of the paired groups were analyzed by a paired Duncan’s t-test. The differences were considered significant when p<0.05. All statistical analyses were performed by using the general linear model procedure of SAS (SAS version 9.2., Cary, NC, USA).

## RESULTS

### Sheep *LIN28B* mRNA sequence and an alternatively spliced isoform

After RT-PCR with the primers B1–B5, five fragments were sequenced and assembled according to the overlap sequences; thus, transcript 1 was obtained, which was 6,630 bp in size and contained 1,396 bp of the 5′ UTR, 744 bp of the entire coding region and 4,490 bp of the 3′ UTR. After RT-PCR with the primers B1–B4 and B6, five fragments were sequenced and assembled according to the overlap sequences; thus, transcript 2 was obtained, which was 5,271 bp in size and contained 13 bp of the 5′UTR, 768 bp of the entire coding region and 4,490 bp of the 3′ UTR. The alignment of the two transcripts indicated that these sequences had different coding regions and 5′ UTRs.

### Sequence homology and phylogenetic relationship

The amino acid sequences were deduced by using DNAman 6.0, the entire coding region of transcript 1 was predicted to encode an- 247 amino acid protein with a calculated molecular mass of 26.67 kDa, and the entire coding region of sheep transcript 2 was predicted to encode an- 255 amino acid protein with a calculated molecular mass of 27.55 kDa. The isoelectric points of *LIN28B* isoform 1 and isoform 2 were 8.78 and 9.08, respectively. The identities of the corresponding protein sequences were 96.08% between the two transcripts, and the transmembrane protein prediction by the TMHMM program showed that the *LIN28B* protein was not a transmembrane protein. The signal peptide prediction by Signalp showed that the *LIN28B* protein was not a signal peptide. Protein sequence analysis in the SMART database revealed that the proteins of the two transcripts contained N-terminal CSDs and a pair of C-terminal retroviral-type CCHC zinc finger domains, and the sequences of the CSDs and CCHC domains were conserved among mammalian species.

**Figure 1:**
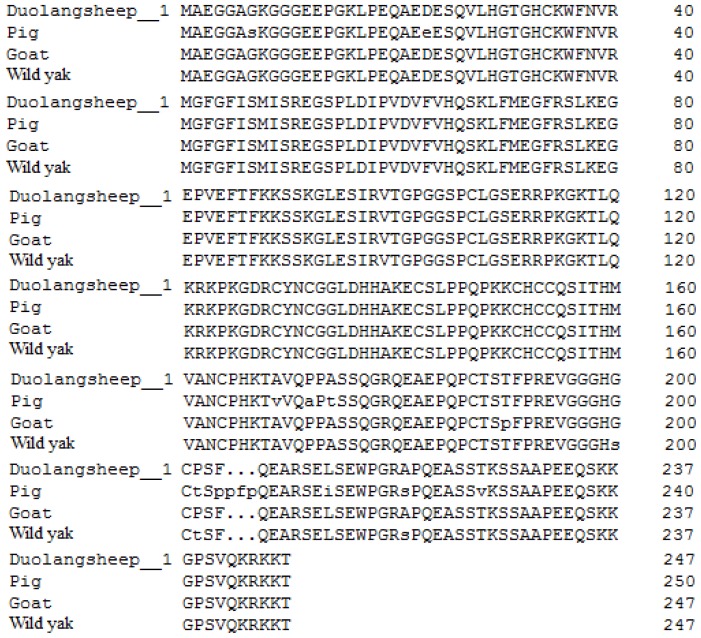
Alignment of the amino acid sequences of the predicted Duolang sheep lin-28 homolog B protein with those of pig (XP_020935097.1), goat (XP_005684673), and wild yak (XP_005901662.1).

**Figure 2:**
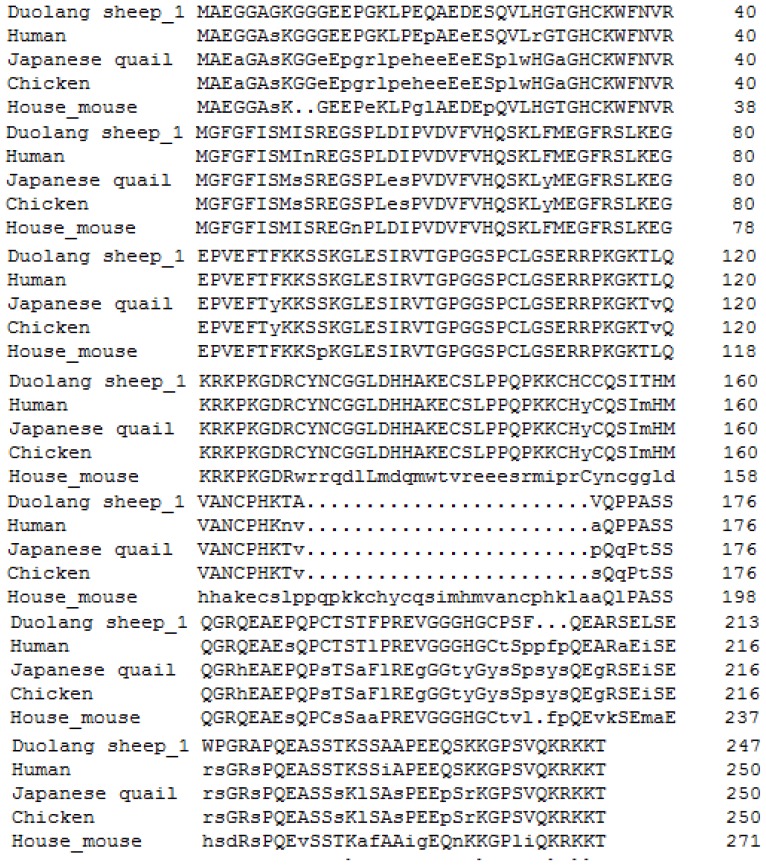
Alignment of the amino acid sequences of the predicted Duolang sheep lin-28 homolog B protein with those of human (AAZ38897.1), Japanese quail (XP_015714194), house mouse (AAZ38894.1), and chicken (AAZ38896.1).

The *LIN28B* isoform1 sequence of Duolang sheep shares 99.60%, 98.78%, and 94.80% identity with those of goat (XP_ 005684673.1), wild yak (XP_005901662.1), and pig (XP_0209 35097.1), respectively (Figure 1). The *LIN28B* isoform1 sequence of Duolang sheep also shares 90.80%, 81.67%, 81.67%, and 76.92% with those of human (AAZ38897.1), Japanese quail (XP_015714194), chicken (AAZ38896.1), and house mouse (AAZ38894.1), respectively (Figure 2).

The phylogenetic relationship of *LIN28B* proteins among different species was investigated by using the Neighbor-Joining method, and the results clearly showed that the two *LIN28B* protein of Duolang sheep were clustered with those of goat, wild yak and pig; the house mouse and human *LIN28B* proteins formed a closely related group, while the Japanese quail and chicken *LIN28B* proteins formed another distinct group.

### Expression profile of the *LIN28B* gene in sheep tissues

**Figure 3:**
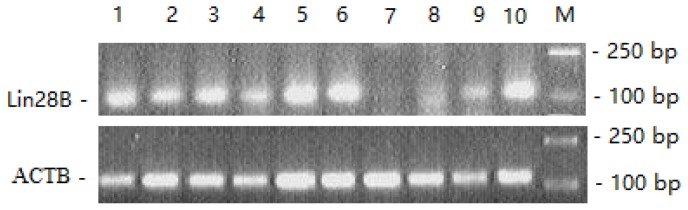
*LIN28B* gene expression profile in ten tissues. 1, Ovary; 2, spleen; 3, uterus; 4, kidney; 5, hypothalamus; 6, pituitary; 7, lung; 8, heart; 9, liver; 10, oviduct; M, DL2000 Marker. *LIN28B*, lin-28 homolog B; *ACTB*, beta-actin.

The expression profiles of *LIN28B* mRNA in ten tissues of Duolang sheep at the prepubertal stage were assessed (Figure 3). Strong expression of *LIN28B* mRNA was found in the hypothalamus, pituitary, ovary and oviduct; moderate expression was found in the spleen, kidney and liver; weak expression was found in the heart; and no expression was found in the lungs.

### Expression levels of *LIN28B* in the hypothalamus, pituitary and ovary of sheep at different pubertal stages

The expression patterns of *LIN28B* in the hypothalamus, pituitary and ovary at three different pubertal stages were detected using real-time PCR.

**Figure 4:**
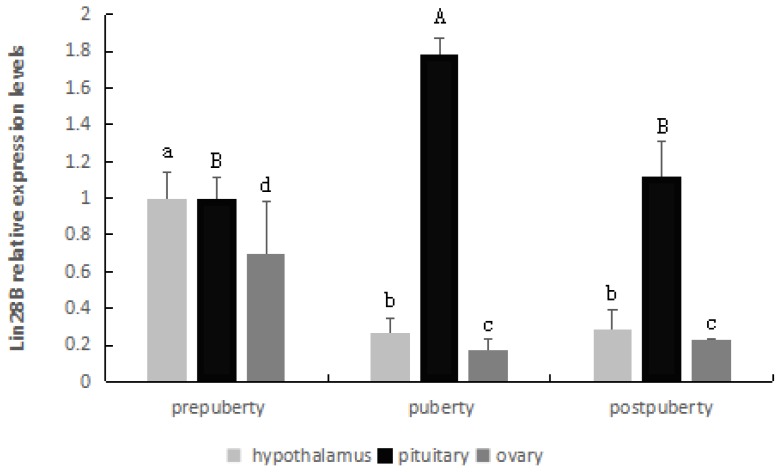
*LIN28B* relative mRNA levels in the hypothalamus, pituitary and ovary. Different letters above the bars in the same tissue indicate significant differences (p<0.05). *LIN28B*, lin-28 homolog B.

As observed in Figure 4, the *LIN28B* gene was highly expressed in the hypothalamus and ovary at prepuberty stages, and its expression was significantly decreased from prepuberty to puberty (p<0.05) but was extremely stable from puberty to postpuberty (p>0.05).

In the pituitary, there was a significantly increased level of mRNA expression from prepuberty to puberty (p<0.05), followed by a statistically significant decrease from puberty to postpuberty (p<0.05).

### Expression levels of sheep *let-7a* and *let-7g* in the hypothalamus, pituitary and ovary across the pubertal transition

To investigate whether the expression of *let-7a* and *let-7g* changes across the pubertal transition, the expression levels of *let-7a* and *let-7g* were detected using qPCR. For *let-7a*, a trend toward increased expression in the hypothalamus and pituitary was observed from prepuberty to postpuberty but did not reach a significant level (p>0.05). The expression of *let-7a* in the ovary showed some changes among different pubertal stages, but there was no regularity and the levels did not reach significance (p> 0.05). Similarly, the expression of *let-7g* in the pituitary gradually increased from prepuberty to postpuberty but did not reach a significant level (p>0.05).

### Protein expression of *Lin28B* in the hypothalamus, pituitary and ovary across the pubertal transition

**Figure 5:**
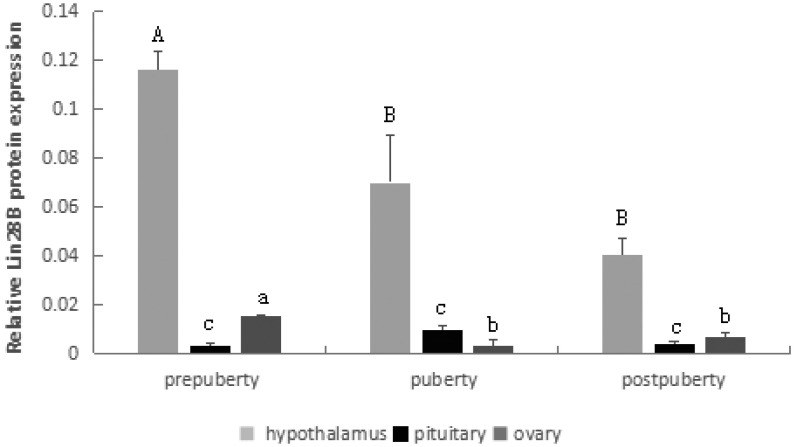
*LIN28B* relative protein levels in the hypothalamus, pituitary and ovary. Different letters above the bars in the same tissue indicate significant differences (p<0.05). *LIN28B*, lin-28 homolog B.

**Figure 6:**
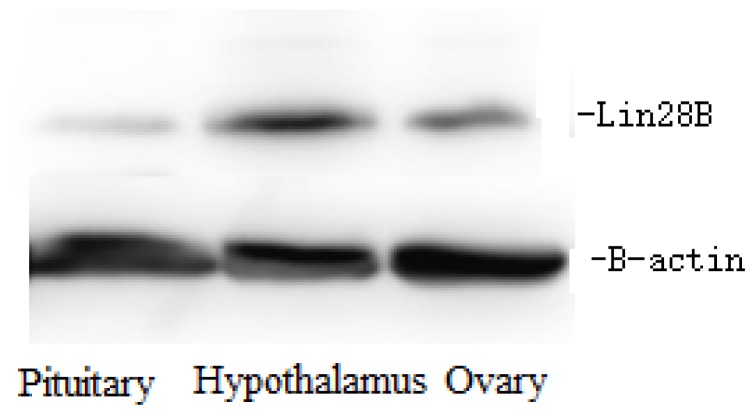
*LIN28B* protein expression in hypothalamus, pituitary and ovary of preubertal sheep. *LIN28B*, lin-28 homolog B.

The protein expression pattern of *LIN28B* in the hypothalamus, pituitary and ovary at three different pubertal stages was detected using Western blotting (Figures 5, 6). As shown in Figure 5, the *LIN28B* protein was highly expressed in the hypothalamus and ovary, but significantly decreased from prepuberty to puberty (p<0.05). Expression of the *Lin28B* protein in the pituitary showed some changes among different pubertal stages, but the levels did not reach significance (p>0.05).

## DISCUSSION

Alternative splicing of eukaryotic transcripts is a mechanism that enables cells to generate vast protein diversity from a limited number of genes [22]. In the present study, the *LIN28B* cDNA sequence of Duolang sheep was cloned and analyzed, and subsequently, we detected two *LIN28B* transcripts that differed in the 5′ UTR and partial coding regions, the results of the present study are consistent with that of goat [23]. The 3′ UTRs of the two transcripts were long and included *let-7* microRNA complementary sites, similar to the human and goat sequences [10,23].

The *LIN28B* protein sequence was highly conserved among mammals [23], and in the present study, the *LIN28B* protein of Duolang sheep showed relatively high homology with those of other mammals. The amino acid sequence of Duolang sheep *LIN28B* transcript 1 shares 99.60%, 98.78%, and 94.80% identity with those of goat, wild yak and pig, respectively, and phylogenetic analysis showed that the phylogenetic tree conformed to evolutionary laws. Similar homology of *LIN28B* was found between goat, human and other known vertebrates [10,23].

The expression patterns of *LIN28B* were detected in several vertebrates [14,17]. *LIN28B* was expressed in many developing tissues and was involved in the biosynthesis of the *let-7* family and the onset of mammalian puberty [24]. Expression of *LIN28B* was highest in the testis, placenta and hypothalamus of human and rats [10,23]. In goats, *LIN28B* was expressed in the hypothalamus, lung and spleen [25]. In the present study, we detected *LIN28B* mRNA expression in most tissues of Duolang sheep; strong *LIN28B* expression was found in the hypothalamus, pituitary, ovary and oviduct; moderate expression was found in the spleen, kidney and liver; weak expression was detected in the heart. The *LIN28B* expression differences in different tissues may exist in different species, and the *LIN28B* gene was mainly expressed in the hypothalamus, pituitary, ovary and testis [26]. From the above results, we concluded that *LIN28B* gene was distributed in the hypothalamus-pituitary-gonadal axis (HPGA), which participates in the onset of puberty regulation. The *LIN28*/*let-7* system including *LIN28A*/*LIN28B* and *let-7* miRNAs, has emerged as putative regulator of puberty [15,17]. In male and female rats, *LIN28B* displayed high hypothalamic expression during the neonatal period, which was markedly decreased during the infantile-to-juvenile transition and reached minimal levels before/around puberty. A similar puberty-related decline was observed for *LIN28B* in the hypothalamus of monkeys [14]. In the present study, *LIN28B* was highly expressed in the hypothalamus at prepuberty stages, but significantly decreased from prepuberty to puberty, and these results were consistent with those of a previous study [14].

In female mice, expression of *LIN28B* decreases in the ovary prior to the onset of puberty, and neither *let-7a* nor *let-7g* levels significantly changed [13]. In the present study, *LIN28B* expression was decreased in the ovaries prior to the onset of puberty, and neither *let-7a* nor *let-7g* levels significantly changed. Additionally, no statistically significant changes were observed in *let-7a* and *let-7g* levels in the hypothalamus and pituitary tissues among different pubertal stages. The present results showed that although *LIN28B* expression was decreased in the hypothalamus and ovary prior to the onset of puberty, neither the *let-7a* nor *let-7g* levels significantly increased. The data suggest that the sheep *LIN28B* effects within the HPG axis may be miRNA independent, which will be an important area for further study.

## CONCLUSION

In summary, the results of the present study showed that *LIN28B* mRNA expression was detected in most of the tissues of Duolang sheep: strong expression of *LIN28B* was detected in the hypothalamus, pituitary, ovary and oviduct; moderate expression was found in the spleen, kidney and liver; weak expression was observed in the heart. *LIN28B* expression was distributed in the HPGA, and although *LIN28B* expression was decreased in the hypothalamus and ovary prior to the onset of puberty, neither *let-7a* nor *let-7b* levels were significantly increased. These data suggest that the sheep *LIN28B* effects within the HPG axis may be miRNA independent and that the *LIN28B* effects within the HPG axis will be an important area for further study.

## Figures and Tables

**Table 1:** Primers used for *LIN28B* gene cloning

Primers	Primer sequence (5′-3′)	Product size (bp)	Annealing temperature (°C)
B1	gggtggtggtgcgtttcagtgt	1,140	56
	ctttggaacggagtggtcttc		
B2	taactactgttgaggaact	1,314	58
	tggaagctgaagggatcaatact		
B3	tactgaagggacgaatacgg	1,400	55
	gtcagaaacatgcagttatga		
B4	acacctttatgctgctccagcct	1,343	57
	cactgtacagtacttgaccat		
B5	acaaagtcacgtgtgctcag	357	55
	ccctctcggcttatcatgga		
B6	ttcttcagaagacaatgag	818	58
	ttgcatgaggtagacttcc		
ACTB-F	ttccagccttccttcctg	109	58
ACTB-R	ccgtgttggcgtagaggt		

*LIN28B*, lin-28 homolog B.
